# Bacterial Dynamics of Wheat Silage

**DOI:** 10.3389/fmicb.2019.01532

**Published:** 2019-07-09

**Authors:** Jitendra Keshri, Yaira Chen, Riky Pinto, Yulia Kroupitski, Zwi G. Weinberg, Shlomo Sela Saldinger

**Affiliations:** Department of Food Science, Institute for Postharvest and Food Sciences, Agriculture Research Organization Volcani Center, Rishon LeZion, Israel

**Keywords:** bacterial diversity, bacterial community, 16S rRNA, silage, aerobic-stability

## Abstract

Knowledge regarding bacterial dynamics during crop ensiling is important for understanding of the fermentation process and may facilitate the production of nutritious and stable silage. The objective of this study was to analyze the bacterial dynamics associated with whole crop wheat silage with and without inoculants. Whole crop wheat was ensiled in laboratory silos, with and without *Lactobacillus* inoculants (*L. plantarum, L. buchneri*), for 3 months. Untreated and *L. plantarum*-treated silages were sampled at several times during ensiling, while *L. buchneri*-treated silage was sampled only at 3 months. Bacterial composition was studied using next generation sequencing approach. Dominant bacteria, before ensiling, were *Pantoea* (34.7%), *Weissella* (28.4%) and *Pseudomonas* (10.4%), *Exiguobacterium* (7.8%), and *Paenibacillus* (3.4%). Exogenous inoculants significantly affected bacterial composition and dynamics during ensiling. At 3 months of ensiling, *Lactobacillus* dominated the silage bacterial population and reached an abundance of 59.5, 92.5, and 98.2% in untreated, *L. plantarum*- and *L. buchneri*-treated silages, respectively. The bacterial diversity of the mature silage was lower in both treated silages compared to untreated silage. Functional profiling of the bacterial communities associated with the wheat ensiling demonstrated that the abundant pathways of membrane transporters, carbohydrate and amino acids metabolisms followed different pattern of relative abundance in untreated and *L. plantarum*-treated silages. Only three pathways, namely base-excision repair, pyruvate metabolism and transcription machinery, were significantly different between untreated and *L. buchneri*-treated silages upon maturation. Lactic acid content was higher in *L. plantarum-*treated silage compared to untreated and *L. buchneri-*treated silage. Still, the pH of both treated silages was lower in the two *Lactobacillus*-treated silages compared to untreated silage. Aerobic stability test demonstrated that *L. plantarum*-, but not *L. buchneri*-supplement, facilitated silage deterioration. The lower aerobic stability of the *L. plantarum*-treated silage may be attributed to lower content of acetic acid and other volatile fatty acids which inhibit aerobic yeasts and molds. Indeed, high yeast count was recorded, following exposure to air, only in *L. plantarum*-treated silage, supporting this notion. Analysis of bacterial community of crop silage can be used for optimization of the ensiling process and the selection of appropriate inoculants for improving aerobic stability.

## Introduction

Ensiling of forage crops is widely practiced worldwide in order to ensure continuous feed supply for ruminant livestock. The ensiling process comprises rapid achievement of anaerobic conditions and anaerobic fermentation of moist forage crops whereby lactic acid bacteria (LAB) convert water-soluble carbohydrates into organic acids, mainly lactic acid, resulting in forage acidification. Accumulation of LAB during the fermentation results in rapid pH decline that enables forage preservation for long time. Basically, ensiling fermentation process comprises four main stages: At initial stage, air is still present between the crop particles and the pH value is above 6.0. At this stage a variety of microorganisms are active. Secondly at the stage of fermentation, air is depleted through the activity of aerobic microorganisms and LAB become the dominant flora. At this stage the pH decreases to a value depending on forage composition and ensiling conditions. After successful fermentation, silage remains stable, as long as no air penetrates into it. At this stage sometimes, lactic acid is converted gradually to acetic acid by hetero-lactic bacteria, such as *Lactobacillus buchneri* (Weinberg and Chen, 2013). The last stage is unloading for feeding, when the silage is re-exposed to air and aerobic microorganisms, mainly yeasts and molds are activated (Weinberg and Muck, 1996). Anaerobic conditions and lactic acid formation, along with other volatile organic acids produced in the course of ensiling, restrict the growth of spoiling microbes e.g., yeasts, molds and other bacteria. LAB play a pivotal role in the ensiling process and many LAB containing additives are commercially available and used to enhance and improve the ensiling process, as well as, aerobic stability (Cai et al., 1998; Xie et al., 2012). LAB species commonly used to facilitate the ensiling fermentation belong to the genera *Lactobacillus*, *Lactococcus*, *Enterococcus*, and *Pediococcus* (Pahlow et al., 2003).

Ensiling has become a common global practice for feed preservation, especially in wet climates where the conservation of dried forage is problematic (Pahlow et al., 2003). The major forage crops worldwide, preserved in the form of silages, include whole-crop corn, alfalfa and various grasses (Wilkinson et al., 2003). Preservation of forages in the form of silages is a well-practiced in the temperate regions of Europe and North America, however, it is attaining a widespread popularity recently in tropical regions, too (Wilkins and Wilkinson, 2015). In Israel with semi-arid climate, wheat, which is grown on winter rains, is the major forage for ensiling. It is harvested in early spring at the milk stage of maturity when yields, ensiling characteristics and nutritional value are the optimum (Ashbell et al., 1997). It is recommended that the whole crop is chopped into 1–2 cm pieces and ensiled at 320–380 g/kg of dry matter (DM) (Weinberg et al., 2009). Ensiling of wheat is advantageous as wheat plant contains high dry matter and water soluble carbohydrate, and it can be grown under wider climatic and soil conditions (Ni et al., 2015). The wheat silages were reported to increase milk production of cows when used as an alternative of corn silages (Wang et al., 2013).

Ensiling is a natural preservation process, which involves complex microbial interactions. A significant effort is required for detailed characterization of the ecology of the microorganisms involved in the ensiling process and associated spoilage (McAllister et al., 2018). Most of the earlier studies related to microbial ecology associated with silages were performed using classical microbiological techniques. Later studies also used molecular techniques, e.g., Real-Time PCR, DGGE, 16S rRNA gene sequencing of isolates, and sequencing of 16S rRNA gene clone library to study the microbial ecology of silages (Stevenson et al., 2006; Parvin and Nishino, 2009, 2010; Pang et al., 2011; McGarvey et al., 2013). The application of next generation sequencing approach in the study of microbial ecology of silages is relatively new and enabled the identification of the microbial taxa associated with grasses (Eikmeyer et al., 2013; Li et al., 2015; Kasmaei et al., 2016; Zhao et al., 2017), oat (Romero et al., 2017), small grains forages (Duniere et al., 2017), soybean (Ni et al., 2017), alfalfa (Ogunade et al., 2018), corn (Kasmaei et al., 2016; Strauber et al., 2016; Zhao et al., 2016; Gharechahi et al., 2017; Keshri et al., 2018; Xu et al., 2018), and drumstick leaves (Wang et al., 2018). Compared to other silages, studies addressing the microbial populations associated with wheat silage are limited. Only few reports are available on the microbial community associated with wheat silages, which are based on cultured isolates and their molecular identification (Bal and Bal, 2012; Ni et al., 2015). In a more recent study, next generation sequencing approach has been applied for the analysis of the bacterial community of mature commercial wheat and corn silages in large bunker silos (Kraut-Cohen et al., 2016). Although, ensiling of wheat is attaining high popularity, in recent years, due to the high nutrient content of wheat and its wider availability (Ni et al., 2015), knowledge on the bacterial dynamics and the metabolic pathways associated with wheat ensiling process is still lacking. Furthermore, knowledge on the interplay between *L. plantarum* and *L. buchneri*, two common silage additives, and the bacterial population of wheat silage, is also missing.

## Materials and Methods

### Ensiling Experiment

Whole-plant wheat harvested at the soft dough maturity stage was chopped to 2 cm pieces and ensiled in 1.5 l anaerobic jars (Weck, Wehr, Germany). Silage treatments included natural microbiome without additives (Untreated) and addition of *L. plantarum* MTD1 (Ecosyl, Stokesley, United Kingdom) (Lp-Treated). The inoculant was applied at a final concentration of ca. 10^6^ CFU/g crop by suspending an adequate amount of the bacteria (based on its concentration in the inoculant product) in 25 ml distilled water and spraying the suspension on 10 kg chopped wheat spread on 2 × 1 m plastic sheeting, followed by thorough mixing. Untreated freshly chopped wheat crops were sampled in triplicates, before ensiling (time 0). Triplicate jars of the untreated and Lp-treated silages were sampled, at 6 h after ensiling, and on days 1, 2, 7, 15, 30, and 90. A similar triplicates of ensiling jars was inoculated with *L. buchneri* (Ecosyl, Stokesley, United Kingdom) (Lb-treated), at a final concentration of ca. 10^6^ CFU/g. Lb-treated- jars were sampled only after 90 days of ensiling in order to compare the effects of *L. plantarum* and *L. buchneri* inoculants on the quality and bacterial community of mature silage. Following sampling on day 90, the silages were subjected to an aerobic stability test for five additional days, as described before (Weinberg et al., 2009). Visual appraisal, production of CO_2_, change in pH and numbers of yeasts and molds served as spoilage indicators.

### Analyses of Silage Characteristics

Dry matter (DM) was determined in triplicate by oven drying for 48 h at 60°C. A mixture 40 g of silages in 360 ml of distilled water was stomached for 3 min (Bagmixer, Interscience, France), the filtered aqueous extract was used for estimation of lactic acid content, ethanol and acetic acid. Lactic acid content was determined by the spectrophotometric method of Barker and Summerson (1941). Ethanol and acetic acid were determined with a gas chromatograph equipped with a semi capillary FFAP (nitroterephthalic acid-modified polyethylene glycol) column (Hewlett Packard, Waldborn, Germany), over a temperature range of 40–230°C. Microbiological evaluation included enumeration of LAB on Rogosa and MRS agar plates (Oxoid, Basingstoke, United Kingdom), yeasts and molds were enumerated on malt extract agar plates (Difco, Detroit, MI, United States) acidified to pH 4.0 with lactic acid. The plates were incubated at 30°C for 3 days.

### Bacterial DNA Isolation

Untreated and inoculant-treated samples, collected at the different time points, were stored at −20°C before further analysis. Isolation of bacterial DNA and purification were performed as described previously (Keshri et al., 2018). Silage samples (10 g) were mixed with 40 ml each of sterilized double-distilled water (SDDW) and vortexed for 2 min. The mixture was filtered using cotton mesh to remove larger plant material and the liquor was centrifuged at 12,800 × *g* for 30 min at 4°C. The supernatant was discarded and pellet was used for microbial DNA isolation using PowerSoil DNA Isolation Kit (Mo Bio Laboratories, Inc., United States), following manufacturer’s protocol. The quality and quantity of the DNA were assessed at A260/280 nm by NanoDrop 2000 spectrophotometer (Thermo Fisher Scientific, United States). The purified DNA was stored at −20°C until used.

### Library Preparation and Sequencing

Bacterial identification was performed using amplicon sequencing of 16S rRNA gene. PCR amplification was carried out to amplify the V4–V5 conserved regions of bacterial 16S rRNA gene sequences using primers 515F and 926R (Caporaso et al., 2011). Library preparation, pooling and sequencing were performed at the DNA Services facility, Research Resources Center, University of Illinois at Chicago. Briefly, genomic DNA was PCR amplified using a two-stage targeted amplicon sequencing protocol (Green et al., 2015). DNA library preparation and sequencing were performed as previously described (Keshri et al., 2018). Sequence data were received as fastq files and submitted to the National Center for Biotechnology Sequence Read Archive under Bio Project accession number PRJNA497711.

### Sequence Data Analysis

Sequence processing was performed using Mothur software, version 1.39.1 (Schloss et al., 2009). Sequence analysis of each combined single fastq file was processed using the Mothur MiSeq SOP accessed on the 3/1/18 (Kozich et al., 2013). The paired-end MiSeq Illumina reads targeting bacterial community from the 57 wheat silage samples were aligned and converted to contigs yielding 3,360,079 reads. Sequences were quality checked and sequences having ambiguous characters, homopolymers longer than 8 bp were removed. Unique sequences and their frequency in each sample were identified and then a pre-clustering algorithm was used to further denoise sequences within each sample. Singletons were removed from the dataset and chimera removal was performed using the UCHIME algorithm (Edgar et al., 2011). Finally, classification of the high quality filtered bacterial sequences (1,869,232) was done by the Mothur version of Bayesian classifier (Wang et al., 2007) with the full length sequences and taxonomy references from NR SILVA database of release 123 (Pruesse et al., 2007). The operational taxonomic unit (OTU) was assigned at 97% identity of the sequences. Rarefaction was performed on each sample to assess sampling adequacy, using a 100 sequence increment. After normalizing the number of sequences in each sample (based on rarefied or subsampled data i.e., the minimum number of remaining sequences in any of the samples; 2200 sequences); alpha diversity was assessed by calculating the richness estimator Chao1 and the Shannon diversity index. Library coverage and species evenness were also calculated. The taxonomic classification and diversity indices of samples from respective replicates were averaged. Beta diversity (variation in community structure between samples) was determined using the same subsampling approach, an index that accounts for proportional abundances of both shared and non-shared OTUs.

### Statistical Analysis

Statistical analysis of silage parameters, relative abundances and diversity indices was performed by Analysis of Variance (ANOVA) and pairs Tukey HSD test using JMP Pro 13.0.0 software (SAS, Cary, NC, United States). Two dimensional non-metric multidimensional scaling (NMDS) plots were generated based on the weighted unifrac distance matrix calculated between different samples using the OTU table with OTU clustering at the species level (Lozupone et al., 2006). Differences in bacterial community composition among the samples were assessed using Unifrac weighted distance metric.

### Functional Profiling of the Bacterial Community Associated With Wheat Silage

Phylogenetic investigation of communities by reconstruction of unobserved states (PICRUSt, release 1.0.0) was used to predict the functional profiling based on the 16S rRNA gene sequences of the bacterial communities associated with the wheat ensiling process (Langille et al., 2013). For this analysis, OTUs were closed-reference picked against the Greengenes database by Mothur (v.1.39.1). The functional taxonomies were normalized, predicted, and categorized according to online protocols of PICRUSt^1^ to generate the predicted KEGGs (Kyoto encyclopedia of genes and genomes) pathways Orthology (KO) classification (Kanehisa et al., 2008). The final predicted metagenome (a biom table), is analyzed with a software package, Statistical Analysis of Taxonomic and Functional Profiles (STAMP) v.2.0.9 (Parks et al., 2014) to test and visualize significant predicted functional differences in bacterial communities between the different ensiling times and type of treatments.

## Results

### Silage Characteristics

**FIGURE 1:**
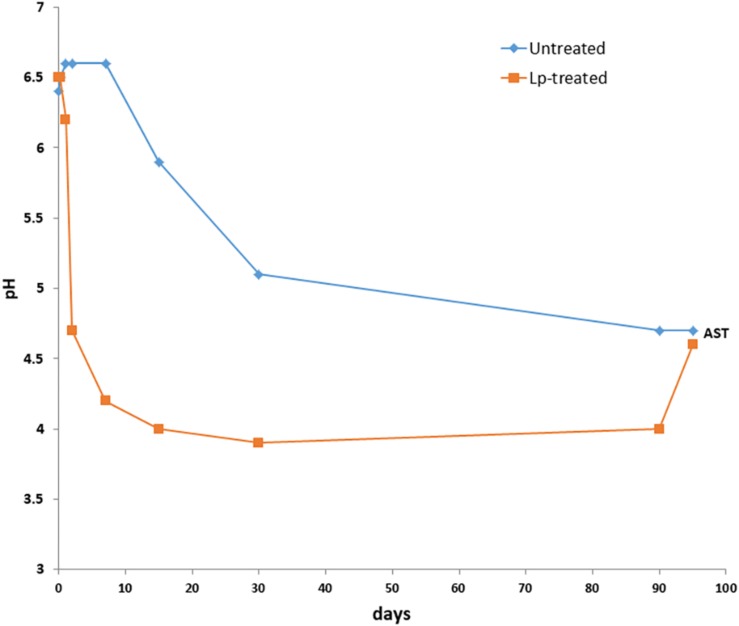
Changes in pH of wheat silage during the course of ensiling with and without *L. plantarum*-supplement. AST denotes 90 days mature silages followed by 5 days aerobic stability test.

The DM content of fresh wheat was 456 ± 6 g/kg and decreased after 90 days of ensiling to 443 ± 4 g/kg. The DM content of Lp- and Lb-treated silages at 90 days, were 455 ± 5 g/kg and 420 ± 9 g/kg, respectively. The initial pH of the freshly chopped wheat samples was 6.5, and it decreased to 4.7 in untreated samples after 90 days of ensiling. However, the pH values of the Lp-treated silages decreased faster and reached a value of 4.2 as early as 7 days and further decreased to pH 4.0 following 15 days of ensiling (Figure 1). The pH of Lb-treated silages was 4.3 after 90 days of ensiling. Microbial, chemical and visual parameters of the silages after 90 d of ensiling as well as after air exposure are summarized in Table 1. Lactic acid content were significantly different among the three silages and was highest in the Lp-treated silages. Acetic acid contents were significantly higher in Lb-treated silages as compared with both untreated and Lp-treated silages. Ethanol concentration was similar in untreated and Lb-treated silages, but was significantly lower in Lp-treated silages. *Lactobacillus* counts were significantly higher in Lb-treated silages and molds were not detected in any of the treatments. Yeast counts were significantly higher in untreated than the Lp-treated silages, and were below the detection level in Lb-treated silages (Table 1). The aerobic stability test indicated that untreated- and Lb-treated silages were more stable than Lp-treated silage, as evident by the higher counts of yeast and molds, as well as CO_2_ produced in the latter case.

**TABLE 1:** Characteristics of the final wheat silages (90 days) and after aerobic exposure for 5 days.

	**Dry matter (g/Kg)**	**pH**	**Lactic acid (g/kg DM)**	**Acetic acid (g/kg DM)**	**Ethanol (g/kg DM)**	***Lactobacillus* counts (log 10 cfu/g DM)**	**Yeast counts (log 10 cfu/g DM)**	**Molds counts (log 10 cfu/g DM)**	**CO_2_ produced (g/kg DM)**	**Visual appearance**
**Final silage**(3 months)										
Untreated	443±4^b^	4.7±0.08^a^	21±0.6^b^	1±0.1^b^	13±1.5^a^	6.7±0.12^b^	6.1±0.1^a^	BDL	nd	Clean with no apparent molds or yeasts
Lp-Treated	455±5^a^	4.0±0.01^c^	32±0.6^a^	2±0.1^b^	7±0.5^b^	5.3±1.34^b^	4.7±0.05^b^	BDL	nd	Clean with no apparent molds or yeasts
Lb-Treated	422±9^c^	4.3±0.01^b^	17±1.2^c^	28±3.8^a^	13±0.8^a^	8.6±0.06^a^	Not detected^c^	BDL	nd	Clean with no apparent molds or yeasts
**After air-exposure** (5 days)										
Untreated	nd	4.7±0.07	nd	nd	nd	6.9±0.35^b^	5.8±2.34^a^	BDL	6.4@0.7^b^	Clean with no apparent molds or yeasts
Lp-Treated	nd	4.6±0.02	nd	nd	nd	7.2±0.2^a,b^	8.0±0.76^a^	5.6@1.55	18.5@0.2^a^	Spoiled and Moldy
Lb-Treated	nd	4.3±0.02	nd	nd	nd	7.6±0.10^a^	Not detected^b^	nd	5.6@0.31^c^	Clean with no apparent molds or yeasts

*BDL, below detection limit of 100 cfu/g DM; nd, not determined; DM, dry matter; ^*abc*^For the same parameters, means followed by different superscripts are significantly different (*P* < 0.05).*

### Bacterial Richness and Diversity During Wheat Ensiling

Overall, 1,869,232 quality filtered 16S rRNA sequences were clustered in to 645 OTUs. The library coverage was more than 99% in all samples. In untreated silage, the richness initially increased and reached its maximum value (91.7 ± 2.1) at 6 h of ensiling (Table 2). The richness then declined and reached its lowest value (33.2 ± 4.07) at day 7, followed by gradual increment up to day 30 (57.2 ± 1.34). A similar initial trend is observed in Lp-treated silage; the relatively high initial richness (70.4 ± 4.64 at 6 h) is declined until day 7 (36.5 ± 1.4). However, the richness then sharply increased to a peak (75.9 ± 1.35) at day 15 and then sharply decline up to day 30 (44 ± 1.17), and stays almost constant until maturation at day 90 (Table 2). In both untreated and Lp-treated silages, richness were almost same at the terminal stage, but the diversity was significantly higher (*P* < 0.05) in case of untreated silages (Table 2). In Lb-treated silages, the richness and diversity at the terminal stage were significantly lower (*P* < 0.05) than both untreated and Lp-treated silages (Table 2).

**TABLE 2:** Diversity indices for bacterial communities associated with Wheat silage samples at different time points.

	**Time point (hours or days)**	**OTUs**	**Chao**	**Shannon diversity index**	**Goods coverage**
Untreated Wheat Silage^*^	0	52.3±0.58	65±3.38	2.3±0.02	0.993±0.002
	6 h	54.7±4.16^a^	91.7±2.1^a^	2.2±0.09^a^	0.99±0.001
	d1	26.7±3.51^a^	44.3±1.24^a^	1.0±0.02^b^	0.995±0.001
	d2	29.7±3.51^a^	47.5±3.01^a^	1.6±0.04^a^	0.993±0.001
	d7	24.3±1.15^a^	33.2±4.07^a^	1.6±0.03^a^	0.996±0.001
	d15	31.7±1.53^b^	38.8±5.2^b^	1.3±0.04^a^	0.996±0.001
	d30	42.7±9.07^a^	57.2±1.34^a^	2.1±0.11^a^	0.994±0.002
	d90	30±2.65^a^	45±1.08^a^	1.8±0.02^a^	0.995±0.002
	AST^*⁣*⁣**^	22.3±2.08^a^	28.2±2.25^a^	1.3±0.01^b^	0.997±0
***L. plantarum* supplemented Silage^∗∗^**					
	6 h	41±5^b^	70.4±4.64^a^	1.8±0.01^b^	0.993±0.003
	d1	37±2^b^	61.1±3.83^a^	1.4±0.09^a^	0.992±0.001
	d2	22.7±3.06^a^	49.1±1.81^a^	0.3±0.02^b^	0.994±0.002
	d7	25.3±3.51^a^	36.5±1.4^a^	0.2±0.04^b^	0.994±0.001
	d15	42.7±3.79^a^	75.9±1.35^a^	0.7±0.08^b^	0.99±0.001
	d30	27.7±3.21^a^	44.0±1.17^a^	0.4±0.04^b^	0.994±0.001
	d90	29.7±4.04^a^	44.5±1.31^a^	0.9±0.02^b^	0.994±0.003
	AST	24.7±8.02^a^	32.2±4.26^a^	1.6±0.08^a^	0.997±0
***L. buchneri* supplemented Silage^∗∗∗^**					
	d90	15±1^b^	20.3±2.27^b^	0.21±0.02^c^	0.997±0
	AST	18.7±4.51^a^	40.9±2.97^a^	0.18±0.03^c^	0.996±0.002

^*^Freshly chopped whole-crop wheat plants ensiled without any inoculants. ^∗∗^Freshly chopped whole-crop wheat plants ensiled with *L. plantarum* at 10^6^ cfu/g crop. ^∗∗∗^Freshly chopped whole-crop wheat plants ensiled with *L. buchneri* at 10^6^ cfu/g crop. ^*⁣*⁣**^AST, denotes aerobic stability test. Indices represent mature silage (d90) following air-exposure for 5 days. ^*ab*^For the same index at respective time points between treatments, means followed by different superscripts are significantly different (*P* < 0.05).

The diversity of untreated silage was maximal (2.3 ± 0.02) at time 0 and lowest at day 1 (1 ± 0.02). The diversity then increased to (1.6 ± 0.04) at day 2, decreased up to day 15 and sharply increased up to day 30 of ensiling. The diversity of Lp-treated silage was lower than the untreated silage at 6 h, and continued to decrease up to day 7 (0.2 ± 0.04). The diversity then increased up to day 15 (0.7 ± 0.08) when it decreased until day 30 (0.4 ± 0.04). A gradual increase in the diversity (from 0.4 ± 0.04 to 0.9 ± 0.02) was observed until day 90. The diversity of untreated silage is significantly higher (*P* < 0.05) than that of Lp-treated silage during the entire ensiling from day 2 to day 90 (Table 2).

**FIGURE 2:**
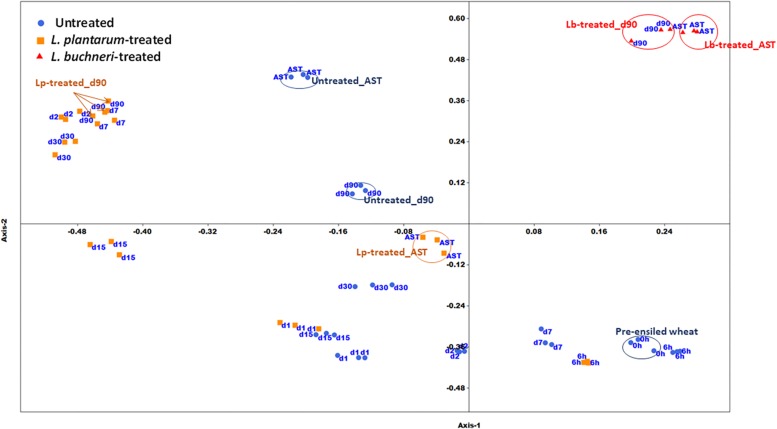
Non-metric Multidimensional Scaling plot of bacterial community composition in untreated and inoculants’ treated silages. AST denotes mature silage samples after aerobic exposure test for 5 days; h and d represents hour and days of ensiling, respectively. Arrows and circles indicate similar treatments/time points.

The two-dimensional NMDS plot depicts different clusters of similar bacterial community composition (BCC) during various ensiling times (Figure 2). A remarkable spatial variation exists in untreated silage between pre-ensiled (time 0) and mature silage (day 90). There is also a significant variation in BCC of mature silage in untreated, Lp- and Lb-treated silage. Exposure of the mature silage to air, for 5 days, resulted in considerable changes in the BCC of untreated and Lp-treated samples, but not in the BCC of Lb-treated silage. Weighted Unifrac metric analysis (Supplementary Table S1), based on OTU abundance, shows no significant changes in BCC of untreated silage from time 0 (fresh chopped wheat) to day 1. Significant changes in BCC was observed during ensiling of both untreated and Lp-treated silages (day 2, 7, 15, 30, 90, and 6 h, day 1, 7, 30, 90, respectively). The BCC of mature silages at 90 day, differed significantly in untreated, Lp- and Lb-treated silages.

### Bacterial Population Dynamics During the Course of Ensiling

**FIGURE 3:**
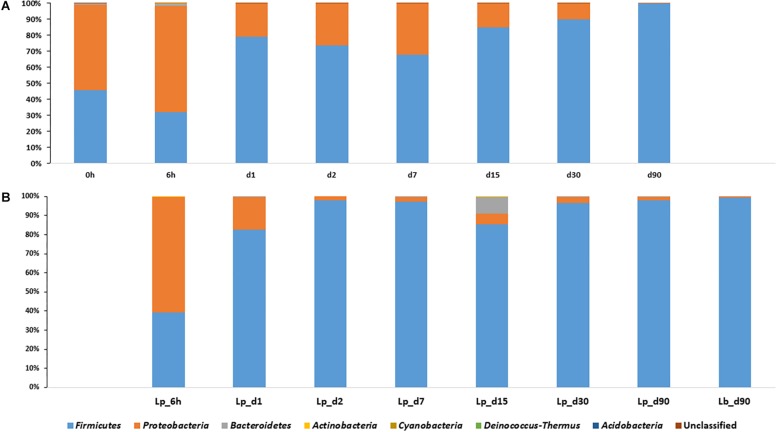
Dynamics of bacterial phyla in course of wheat ensiling without inoculant **(A)**, and with *L. plantarum*- and *L. buchneri*-supplement **(B)**.

*Proteobacteria* were the dominant population in pre-ensiled wheat (time 0), with an abundance of 53.6%, and their abundance decreased during ensiling down to 0.12, 2, and 0.7% in untreated, Lp- and Lb-treated silages, respectively. The *Firmicutes* dominated the mature silage population with an abundance of 99.8, 97.9, and 99.2% in untreated and Lp- and Lb-treated silages, respectively (Figure 3). Overall, the *Firmicutes* and *Proteobacteria* were the most abundant phyla, at any time point during ensiling, and constituted together more than 98% of relative abundances.

**FIGURE 4:**
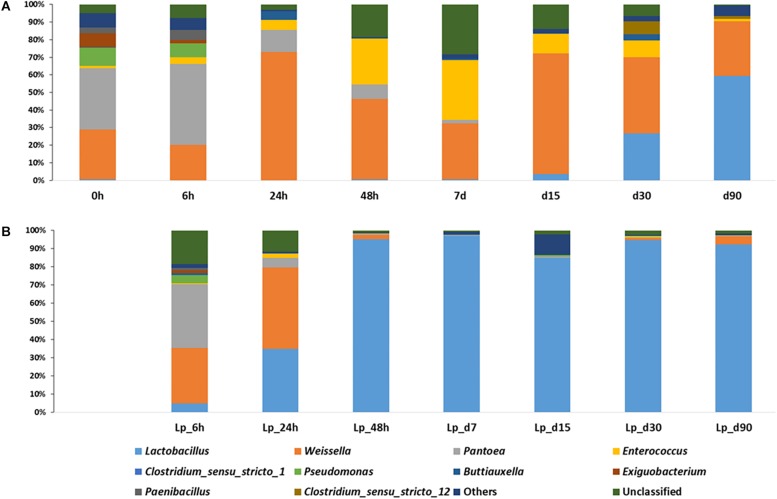
Dynamics of abundant bacterial genera during the course of ensiling in **(A)** untreated silage and **(B)** *L. plantarum-*treated silage. Ten most abundant genera are presented, whereas all other identified genera were presented as “Others.”

The dominant genera in freshly chopped, pre-ensiled wheat plants, were *Pantoea* (34.7%), *Weissella* (28.4%), *Pseudomonas* (10.4%), *Exiguobacterium* (7.4%), and *Paenibacillus* (3.4%). After 6 h of ensiling, the abundance of *Pantoea* increased to 46.3%, but then decreased continuously during silage maturation and at the terminal stage of 90 days they represented only 0.02% of the overall population. The relative abundance of *Weissella* kept on fluctuating during the course of ensiling. *Pseudomonas* decreased continuously during ensiling and was not found in mature silage. *Enterococcus* increased significantly at day 7 where it represented 33.9% of relative abundance and then decreased to reach 1.8% in mature silage. *Lactobacillus* was initially presented at very low abundance (0.53%), which decreased after 6 h of ensiling to 0.09%, but then increased continuously up to 59.5% relative abundance in mature silage (Figure 4A).

In Lp-treated silage, *Pantoea* remained the most abundant genus (34.9%) after 6 h of ensiling, then it decreased during ensiling up to 0.16% at the terminal stage. *Weissella* abundance was increased at 6 h (30.5%) and on day 1 (44.7%), then declined and in the mature silage it was represented by 4.4% of the bacterial populations. Similar to untreated samples, *Pseudomonas* decreased during ensiling and was not found at 90 days of ensiling. The relative abundance of *Enterococcus* was maximum at day 1 (2.3%), and then decreased to reach 0.28% at 90 days. *Lactobacillus* species were in low abundance (4.8%), at 6 h of incubation, and rapidly increased to 34.8% at day 1, and at day 2, they already encompassed more than 95% of the population. Their relative abundance was maximum at day 7 (96.8%), and dropped to 92.4% at 90 days (Figure 4B).

### Effect of Inoculants on Bacterial Composition in Mature Silage

**FIGURE 5:**
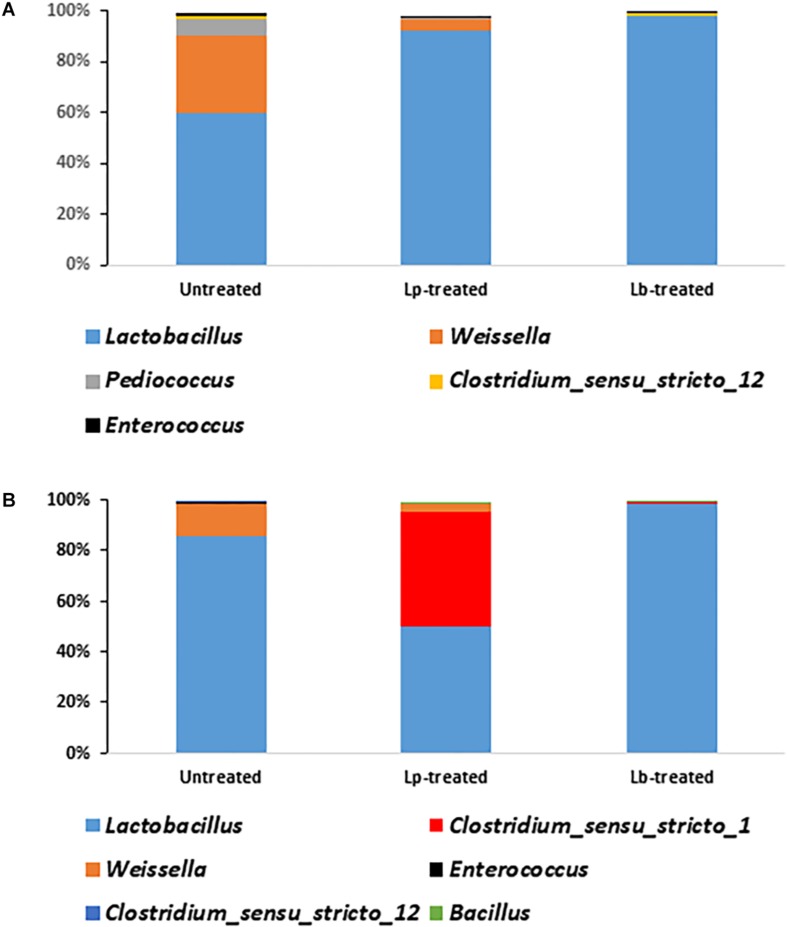
Dominant bacterial genera in wheat silages **(A)** after 3 months of ensiling and **(B)** after aerobic stability test. Untreated denotes wheat silage ensiled with no supplement, Lp-treated and Lb-treated denotes wheat silage ensiled in the presence of *L. plantarum*, and *L. buchneri*, respectively. Only genera with relative abundance > 0.1% are presented.

There was a significant difference in the relative abundance of *Lactobacillus* sp. between Lp- and Lb-treated and untreated silages at the terminal stage of ensiling (*P* < 0.05) (Figure 5A). Although *Lactobacillus* was the most abundant genus in all treatments, it encompassed only 59.5% of the population, in untreated silage, followed by *Weissella* (31%), *Pediococcus* (5.8%), *Clostridium_sensu_stricto_12* (1.6%), *Enterococcus* (1.1%). In contrast, the abundance of *Lactobacillus* in treated silages was much higher. In Lp-treated silage, *Lactobacillus* species represented 92.4% of the entire bacterial population followed by *Weissella* (4.4%) and *Enterococcus* (0.28%). In Lb-treated silages, *Lactobacillus* were the most dominant genus covering 98.2% of the population followed by *Clostridium_sensu_stricto_12* (0.7%) and *Enterococcus* (0.1%) (Figure 5A). Only 21 genera were detected in untreated mature silage, while 31 and 38 genera were identified in Lp- and Lb-treated silages, respectively (Supplementary Table S2).

### Bacterial Dynamics During Aerobic Stability Test

To test silage stability, Lp-, Lb-treated and untreated mature silages, were air-exposed for 5 days and their quality parameters were analyzed. The bacterial richness of the untreated and Lp-treated silages slightly decreased during exposure to air, whereas it increased in Lb-treated silage (Table 2). The diversity decreased in the untreated and Lb-treated silages, while it increased in Lp-treated silage (Table 2). After exposure to air, *Lactobacillus* remained the most dominant population in all silages (Figure 5B). In untreated silage, *Lactobacillus* significantly increased from 59.5 to 85% (*P* < 0.05), while in Lb-treated silage, its abundance remained constant (98%) after air exposure. A significant change was observed in Lp-treated silages, where *Lactobacillus* dropped from 92.4% in mature silage to 49.8% after air-exposure, whereas the genus *Clostridium_sensu_stricto_1* increased from 0.03 to 45.3%. Other prevalent genera that were detected, in air-exposed untreated silage were *Weissella* (12.7%), *Enterococcus* (0.5%) and 12 other genera with relative abundance below 0.05%. In total, 16 and 17 genera were identified in untreated and Lp-treated silages, respectively, while 30 genera were detected in Lb-treated silages (Supplementary Table S3).

### Predicted Function of Bacterial Community Associated With Ensiling

Understanding the metabolic pathways during crop’s ensiling is important for the optimization of the process. In order to gain knowledge regarding potential metabolic pathways that are involved in ensiling, the bacterial community data was analyzed by PICRUSt. Overall a total of 268 metabolic pathways were predicted in all silages using the Kyoto Encyclopedia of Genes and Genomes (KEGG). The most abundant predicted pathways, in pre-ensiled wheat, were (general) transporters (7.7%), ABC transporters (4.3%), DNA repair and recombination proteins (2.5%), two-component systems (2.4%), purine metabolism (2.1%), secretion systems (2.1%), transcription factors (2%), other ion-coupled transporters (1.7%), bacterial motility proteins (1.6%) and ribosome (1.6%). Variations in the abundance of the pathways were observed during ensiling and after aerobic exposure in both untreated and treated silages (Supplementary Table S4), and were visualized using heat map plot (Supplementary Figure S1).

**FIGURE 6:**
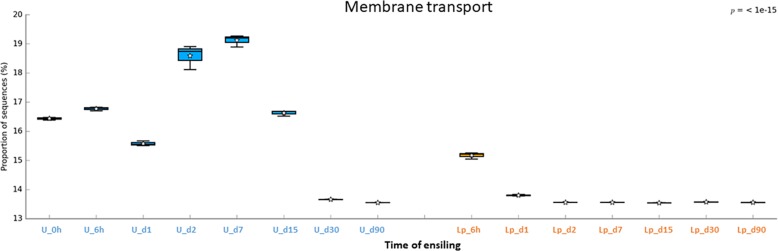
Functional prediction of membrane transporters during untreated and Lp-treated wheat ensiling using PICRUSt.

**FIGURE 7:**
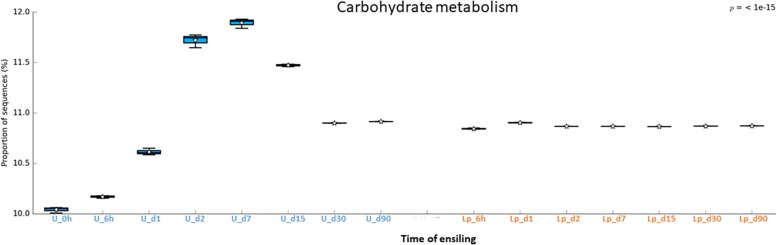
Functional prediction of carbohydrate metabolism during untreated and Lp-treated wheat ensiling using PICRUSt.

**FIGURE 8:**
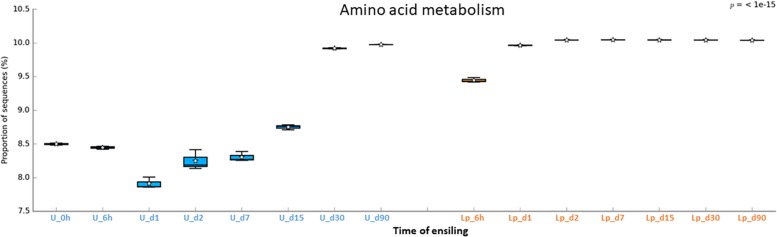
Functional prediction of amino acid metabolism during untreated and Lp-treated wheat ensiling using PICRUSt.

The abundant pathways of membrane transporters (general and ABC transporters) and metabolisms (carbohydrate and amino acids) followed different pattern of relative abundance in untreated and Lp-treated silages (Figures 6–8). Comparison of mature silages (d90), showed 32 significantly different pathways among untreated, and Lp-treated silages, which have more than 0.001% of difference in their relative abundance at d90. These pathways belonged to the functions of membrane transporters, cell motility, carbohydrate-, amino acids-, energy-, nucleotide-, terpenoids and polyketides- metabolisms, glycan biosynthesis and metabolisms, signal transduction, folding, sorting and degradation, replication and repair, and translation (Supplementary Figure S2). Comparison of Lb-treated to non-treated control after 90 days showed only 3 pathways that were significantly different (>0.001%) between the two treatments (Supplementary Figure S3). The pathways belonged to base excision repair, pyruvate metabolism and transcription machinery.

## Discussion

Wheat is a major crop used for silage in Israel and China (Weinberg et al., 2009; Ni et al., 2015). While recent studies have analyzed the microbiome dynamics during ensiling of several other crops (Keshri et al., 2018; McAllister et al., 2018), the present study is the first to analyze the dynamics of wheat bacterial community in the course of ensiling, as well as during aerobic stability test. The data presented contribute basic knowledge regarding the bacterial players associated with high-quality wheat ensiling process, as well as regarding the effect of LAB-supplements on bacterial composition.

The DM content of the wheat used for silage preparation was above the recommended DM content 320–380 g/kg for optimal ensiling of wheat (Ashbell et al., 1997). However, since wheat for silages is harvested in Israel in spring, with abrupt changes in weather from cool to hot and dry, sometimes the wheat dries more than planned before the onset of ensiling (Weinberg et al., 2009). The high DM content of the wheat in the current study provided an opportunity to elucidate changes in bacterial community composition in a slow ensiling fermentation, and determine the effects of LAB inoculants under such conditions. The ensiling of the untreated wheat silages was rather slow and the final pH of untreated silage was 4.7, which is similar to the pH of alfalfa silage with a similar DM content (450 g/kg) (Santos and Kung, 2016). Wheat ensiling, with lower DM content (301 g/Kg), also resulted in different pH in untreated (pH 4.4) and Lp-treated (pH 3.8) silages after 56 days of ensiling (Chen and Weinberg, 2014). The pH of the Lp-treated wheat silages, decreased faster and to lower values of the pH. This is in accordance with the previous study performed on wheat ensiling where LAB inoculants have significant effect on decrease of pH and correlated to DM content (Pieper et al., 2011). It is possible that the high DM content of the untreated silage has limited the multiplication of the indigenous LAB of the wheat. In contrast, supplementation of the silage with exogenous *L. plantarum* inoculant overcame this effect and resulted in significantly faster and improved ensiling. The considerable fermentative activity of the inoculated LAB was also observed in wheat ensiling with high (884 g/kg) DM content (Pieper et al., 2011). DM content is an important parameter in the ensiling process, which may significantly influence the BCC during ensiling with and without inoculants (Pieper et al., 2011), as is evident by BCC of our ensiled wheat silage and, pH dropping that followed different pattern in both cases. Unlike wheat, corn ensiling with rather lower DM (381 ± 7.5 g/kg) resulted in a similar pattern of pH drop in both untreated and Lp-treated silages (Keshri et al., 2018). It is possible that the different patterns of pH change in untreated wheat and corn silages evolved from differences in the moisture and subsequent water activity of the two crops.

The application of NGS to gain insight into the ensiling process is relatively new and has already provided a better understanding regarding the complexity of the microbial ecology of ensiling, role of natural epiphytes and inoculants in high quality silage production (McAllister et al., 2018). The NGS approach is advantageous over culture-dependent and – independent techniques (Bokulich and Mills, 2012; Ni et al., 2017) and to the best of our knowledge, it has been used so far in only five studies to investigate bacterial population dynamics during ensiling. Four studies dealt with corn silages (Zhao et al., 2016; Xu et al., 2017, 2018; Keshri et al., 2018) and one analyzed Manyflower silvergrass silage (Li et al., 2015). In the present study NGS approach was applied for the first time to analyze bacterial population dynamics in wheat silage with and without *Lactobacillus* inoculants used as additives in commercial ensiling processes in order to facilitate the ensiling process (Weinberg et al., 1999; Filya, 2003; Jatkauskas et al., 2013; Arasu et al., 2014).

Nearly identical bacterial populations were observed in triplicate ensiled laboratory silos, at each time point, supporting the data robustness of the silos model, similar to previous reports regarding corn ensiling (Ni et al., 2017; Keshri et al., 2018). In untreated silage, the richness initially increased and reached its maximum value at 6 h of ensiling. This initial dynamics may reflect the multiplication of aerobic bacteria initially when oxygen was still available. The diversity of Lp-treated silage at 6 h was lower than the untreated silage, probably because of the high concentration of the added *L. plantarum*. OTUs richness and bacterial diversity were observed to fluctuate during the first weeks of ensiling with different patterns in untreated and Lp-treated silages. In the untreated silage, the reduction in OTU richness might be related to exhausting of available sugars consumed by yeasts, aerobic and facultative aerobic bacteria during the initial aerobic phase of the ensiling process. From d7 to d30, the richness increased, likely due to the development of LAB populations under the increased acidic conditions. In Lp-treated silages, *Lactobacillus* dominated from d2 of ensiling and imposed a rapid decrease in pH, which apparently inhibited the growth of acid-susceptible bacteria and facilitated the multiplication of selected acid-tolerant LAB population. Indeed, more genera were detected from d7 to d30 in control compared to Lp-treated silage.

Lower diversity in LAB treated silages as compared to untreated silages were also reported in rice straw and corn ensiling (Wang et al., 2016; Keshri et al., 2018), which can be attributed to the inhibitory effect of the LAB population upon the multiplication of other microbes, before the development of the indigenous LAB population (Keshri et al., 2018). A decrease in bacterial diversity upon ensiling was also observed in other crop silages and ensiling times using NGS (Eikmeyer et al., 2013; Kasmaei et al., 2016; Ni et al., 2017; Romero et al., 2017; Duniere et al., 2017).

The indigenous bacterial composition present on silage plants is determined by the type of the crop (Kasmaei et al., 2016; Duniere et al., 2017). In freshly chopped corn, *Proteobacteria* were reported to be the dominant population present at an abundance of 82.6%, and *Firmicutes* encompassed only 14.7% of the population (Keshri et al., 2018), while in pre-ensiled oat *Firmicutes* were the most abundant (83.6%), whereas *Proteobacteria* occupied only 16.2% of the population (Romero et al., 2017). In the case of wheat, the two phyla were represented in comparable abundance (53.5 and 45.7%) in the freshly chopped crop (time 0) and showed antagonistic relationship during ensiling.

Determination of plants bacterial populations may sometimes be biased by the methodology used (Lebeis, 2017). However, Since, the bacterial populations in freshly chopped corn (Keshri et al., 2018) and oat (Romero et al., 2017) plants was analyzed by the same NGS approach using Illumina’s MiSeq methodology, it is likely that the nature of the dominant phyla in each crop is attributed to the type of crop. Still it is possible that the microbial population on the plant might have been influenced by the ecology of the field, too (Berlec, 2012). Regardless of the nature of crop, and the methodology used to determine bacterial community, *Firmicutes*, and more specifically LAB were reported to dominate silages (McEniry et al., 2008; Parvin and Nishino, 2009; Bao et al., 2016; Duniere et al., 2017; Ni et al., 2017; Romero et al., 2017; Keshri et al., 2018), suggesting that anaerobic and low-pH conditions developing during ensiling, favor their multiplication and persistence in the course of silage maturation.

The *Firmicutes* genus mostly associated with mature silage is *Lactobacillus* (Eikmeyer et al., 2013; Li et al., 2015; Keshri et al., 2018; Xu et al., 2018). Species of this genus reached 59.5% relative abundance in untreated, mature wheat-silage, and encompassed a much higher abundance in Lp- and Lb-treated silages (92 and 98%, respectively). Notably, *Lactobacillus* spp. were comparatively more pronounced in the case of mature corn silage, where they encompassed more 94% of relative abundance in both untreated and Lp-treated silages (Keshri et al., 2018). This difference in response to Lp application between corn and wheat could be attributed to the differences in the DM content between the two crops. Indeed, in the corn ensiling experiment there was no difference in the rate of decrease of pH between untreated and Lp-treated silages, whereas in the dry wheat the pH decrease rate differed significantly between the control and Lp-treated silages. Using NGS approach, a high relative abundance of *Lactobacillales* was also reported in grass, wilted rye grass (Eikmeyer et al., 2013), and corn silage (Ni et al., 2017). Dominance of LAB (>80% of *Lactobacillaceae*) was also observed in three out of four good-quality wheat silages taken from commercial bunker silos, whereas less than 10% of *Lactobacillus* was present in the fourth sample (Kraut-Cohen et al., 2016). Thus, laboratory silos, used in this study, seem to provide a good model for studying the dynamics of bacterial community during commercial ensiling.

It is surprising that at the end of ensiling, fewer genera were detected in untreated mature silage compared to treated silages, since the high lactic acid content and the low pH conditions should have limited bacterial growth to acidophilic or acid-tolerant genera. The reason for this finding is not clear. Notably, in corn ensiling, an opposite trend was observed with fewer genera (22) found in Lp-treated silage as compared to the untreated silages (54 genera) after 3 months of ensiling (Keshri et al., 2018). Future studies using different crop should be employed to verify whether this phenomenon is unique to wheat silage or not.

In order to predict the potential metabolic pathways involved in ensiling of wheat crop, metagenomics functional prediction analysis was performed. The relative abundance of pathways was evaluated using KEGG classification (levels 1–3). Overall, the relative abundance of pathways (at level 3) varied in untreated compared to Lp-treated silages. Low variation of metabolic pathways is observed on days 30 and 90 in the untreated silage, as well as during all time points in the Lp-treated silage. These findings are likely related to the high dominance of distinct *Lactobacillus* population under these conditions. Notably, the most abundant predicted pathway in both silages was transporters, the 2nd most abundant pathway/function was “General function prediction only, Unclassified,” which mainly consists of “poorly characterized” pathways. This finding underlines the importance of yet unknown pathways/functions in the ensiling process.

The putative abundance of transporters was much higher in untreated silage until d15, compared to Lp-treated silage. To the best of our knowledge, such high abundance of transporters during ensiling was not reported before. These findings may infer a commensal relationship among the microbial populations in the untreated silage. Indeed, the initial ensiling stages are characterized by the presence of heterogeneous aerobic and facultative anaerobic populations, which consume the oxygen trapped in the chopped forage and increase CO_2_ level. During these stages, metabolic byproducts released by some of the populations may be taken by others, using transporters, in order to obtain essential nutrients for energy and growth. As the pH level decreases up to d15, the silage population becomes more homogeneous and dominated by LAB. Consequently, less transporters are required. In contrast, in the case of Lp-treated silage, the pH level decreases sharply, as early as d2, and *Lactobacillus* dominated the population, so less transport function was required.

A similar phenomenon was observed regarding carbohydrate metabolism. In the Lp-treated silage, a constant level of carbohydrate metabolisms observed throughout the ensiling stages, which apparently reflects the metabolism of the dominant *Lactobacillus* population. In contrast, carbohydrate metabolisms increased in untreated silages until d15 to a much higher levels, and then reduced at d30 and remained constant until d90, at a level comparable to that of *Lactobacillus* treated silages. This is possibly due to the high initial activity of the heterogeneous microbial population at the early ensiling stages. Once oxygen was consumed, and pH level reduced, LAB became the dominant population and anaerobic fermentation took place at d30 through d90.

Unlike transporters and carbohydrates metabolism, the amino acid metabolism was higher in Lp-treated as compared to untreated silages until d30. The low metabolism might reflect the capacity of the initial microbial populations, in the silage, to synthesize amino acid *de novo*. In contrast, as LAB do not synthesize all their essential amino acid, they rely on proteolytic systems to provide essential amino acids for their growth (Novik and Savich, 2019). Consequently, the observed dynamic of amino acids metabolism in both Lp-treated and untreated silage, may reflect the metabolism in the dominant population throughout the ensiling process.

Silage are inevitably exposed to air when used for feeding, leading to undesirable microbial multiplication, decreasing nutritive values, and increasing risk of potential pathogens (Driehuis and Oude Elferink, 2000). Aerobic spoilage is primarily initiated by lactic acid assimilating yeasts, which can thrive well in high lactic acid containing silages (Moon and Ely, 1979). Breakdown of lactic acid leads to rise in pH, eventually providing better conditions for multiplication of opportunistic aerobic bacteria (e.g., *Bacilli*, *Listeria monocytogenes*) and molds (e.g., *Aspergillus*, *Fusarium*, *Pencillium*), which degrade silages subsequently to severe extent (Woolford, 1990; Pahlow et al., 2003).

Aerobic exposure of mature wheat silage for 5 days (aerobic stability test), resulted in a rapid deterioration of Lp-treated silage compared to untreated and Lb-treated silage. A possible explanation for the lower aerobic stability of Lp- versus Lb-treated silage is that *L. plantarum* is a homofermentative bacterium, which produce fermentation products high in lactic acid, and cannot produce enough volatile fatty acids to inhibit yeasts and molds (Weinberg et al., 1993). Indeed, the *L. plantarum* additive used in this study was already shown to enhance spoilage of wheat silage as compared to control (untreated) silage (Chen and Weinberg, 2014) and others have also reported that the usage of homolactic inoculants reduced the aerobic stability of wheat silage ensiled at bunker silos scale (Oliveira et al., 2018).

Unlike Lp, *L. buchneri* is a heterofermentative species, which produce a mix of products, and is able to ferment lactic acid to acetic acid (Weinberg et al., 1993; Weinberg et al., 1999; Filya, 2003), resulting in a higher content of acetic acid, which is a better inhibitor of yeast and mold (Weinberg et al., 1993; Filya, 2003). Indeed, mature Lb-treated silage contained 14-fold more acetic acid than Lp-treated silage, and no detectable yeasts. In support of the role of *L. buchneri* is stabilization of the mature silage, most LAB species isolated from untreated, 30 days-old ensiled wheat silage, were heterofermentative (Ni et al., 2015).

At the terminal stage of ensiling (day 90), a significantly higher content of lactic acid was evident in Lp-treated compared to untreated silage. This may set the stage for the emergence of *Clostridia*, in LP-treated silage, which can utilize lactic acid as substrate (Borreani et al., 2018). Indeed, after aerobic exposure, *Clostridium*_sensu_stricto_1 has emerged as the second most abundant genus. It is likely that the faster deterioration of this silage was associated with the emergence of this genus. In support of this notion, anaerobic spore-formers were previously linked to spoilage of silage, likely because they are capable of converting lactic acid to acetic- and butyric acids, which further contribute to quality deterioration (Vissers et al., 2007; McEniry et al., 2008; Li and Nishino, 2011). While, aerobic stability test, is considered as an indicative test for predicting the stability of silage in commercial bunkers, data obtained from air-exposed silage in commercial wheat silage bunkers, revealed low abundance of *Lactobacillaceae* (<2%), unlike the laboratory silos’ model, and were dominated by *Bacillaceae*, *Thermoactinomycetaeae grp I* and *Enterobacteriaceae* (Kraut-Cohen et al., 2016). These differences may be attributed to the more complex environmental factors exist in the field, as well as to the period of air exposure.

Predicted pathways that might be associated with aerobic stability were analyzed by PICRUSt. Pathway analysis using KEGG classification at level 2, did not yield significant differences between untreated and Lp-treated silage, consequently, KEGG pathway analysis at level 3 was employed. A total of seven pathways were significantly different between untreated and Lp-treated silages after aerobic exposure. In untreated silages, pathways for ethylbenzene degradation, D-Alanine metabolisms, terpenoid backbone synthesis and ion channel systems were more abundant, while in Lp-treated silages two component systems, novobiocin biosynthesis were higher. It is possible that production of antibiotics restricted the multiplication of some bacterial communities, which are competing for substrates with other bacterial and fungal communities. Since, the PICRUSt data are based on bacterial 16S sequences, further metagenomics studies should examine if antibiotics biosynthesis pathways are indeed expressed during ensiling in order to support this notion. Future studies based on metagenomics, covering both bacterial and fungal communities will be required for a more comprehensive understanding of the various metabolic networks and interactions present in the silage microbiome.

PICRUSt has been recently applied in pathways prediction also in maize (Gharechahi et al., 2017) and barley silages (Liu et al., 2019). The PICRUSt analysis, using KEGG level 3 classification, confirmed the changes in metabolic pathways after LAB addition during barley ensiling (Liu et al., 2019). It was anticipated that higher abundance of carbohydrate (Glycolysis, Fructose-and-mannose-, Pyruvate-metabolism, Pentose phosphate pathway) and amino acids (Alanine-aspartate-and-glutamate-metabolism) pathways were responsible for the better aerobic stability of LAB-treated barley silages compared to control. Yet, in our study, the Lp-treated wheat silage was less stable than untreated control. The different findings regarding the aerobic stability of wheat and barley silages, may be due to the nature of the LAB inoculants used, as well as to the nature of the ensiled crop. While in our study, a single species (Lp) was used, in the case of barely, three species, *L. plantarum*, *L. casei*, and *L. buchneri* were added at a ratio of 2:1:1 (Liu et al., 2019). In another study, pathway prediction was employed in untreated maize ensiling. Abundance of pathways for nitrotoluene and styrene degradation, cell motility and secretion, and arginine- and proline- metabolism were reduced during ensiling, while pathways for ethylbenzene, naphthalene and xylene degradation, biosynthesis of glycosphingolipid and bile acids were increased (Gharechahi et al., 2017). The changes in pathways abundance of untreated wheat silage during ensiling showed similarity in various pathways/functions (cell secretion and motility, arginine- and proline- metabolisms, ethylbenzene-, naphthalene-degradation, biosynthesis of glycosphingolipid and bile acids) with maize silage (Gharechahi et al., 2017). Nevertheless, the abundance of pathways of nitrotoluene-, styrene-, ethylbezoate- and xylene-degradation, showed an opposite trend until day 30 of ensiling, and showed subsequently similar level of abundance until 90 days.

The Lb-treated silage, which was rather stable following aerobic exposure, differed from non-treated silage at day 90 in three pathways that belonged to base excision repair, pyruvate metabolism and transcription machinery. Notably, a higher abundance of pyruvate metabolism, among few other pathways, was also observed in LAB-treated barley silage, and was suggested as a possible reason for enhancing of silage stability (Liu et al., 2019).

While taxonomic analyses were based on the more updated SILVA database, the PICRUSt profiling was based on the taxonomic IDs obtained from Greengenes database. Consequently, changes in the bacterial composition based on the two databases might affect the obtained PICRUSt results. However, comparison of the main OTUs, obtained according to both databases, showed high similarity in the taxonomic assignments (data not shown).

Finally, it should be noted the data obtained from functional metagenome prediction must be analyzed cautiously, since the prediction relies on genomic data and 16SrRNA amplification, rather than on direct mRNA measurements (Gharechahi et al., 2017). Therefore, further studies should employ metagenomics and transcriptomics in order to decipher the important metabolic pathways of both bacteria and fungi involved in the ensiling process of various crops.

## Conclusion

The bacterial community of fresh whole crop wheat was found to be dominated by *Pantoea*, *Weissella*, *Pseudomonas*, *Exiguobacterium*, and *Paenibacillus*. During wheat ensiling with and without LAB-inoculant, the bacterial community shifted dramatically and the terminal silage was dominated mainly by *Lactobacillus*. There was a substantial difference in the abundance of *Lactobacillus*, between untreated and LAB-supplemented silages. Exogenous *L. plantarum* significantly affect the bacterial composition and dynamics during ensiling and resulted in a higher dominance of LAB in the mature silage and a higher content of lactic acid. Notably, addition of *L. plantarum*, but not *L. buchneri* reduced aerobic stability of the wheat silage. The lower aerobic stability of the *L. plantarum*-treated silage may be attributed to the different fermentation pathways in the two LAB inoculants. PICRUSt analysis provided knowledge regarding the role of transporters and carbohydrate metabolism in the initial stages of ensiling until stabilization of the bacterial populations. Since, ensiling is an uncontrolled fermentation process, depending on multiple parameters, the ability to monitor the bacterial dynamics, through the process, holds promise for a better understanding and improvement of the silage quality.

## Author Contributions

SSS conceived and coordinated the study and edited the manuscript. JK, ZW, and SSS designed the experiments. JK, YC, RP, and ZW ensiled the wheat crop and collected the samples. JK carried out the molecular biology and bioinformatics analyses. YC and YK carried out the microbiological and biochemical analyses. JK and ZW drafted the manuscript with input from all co-authors. All authors read and approved the final manuscript.

## Conflict of Interest Statement

The authors declare that the research was conducted in the absence of any commercial or financial relationships that could be construed as a potential conflict of interest.

## Footnotes

1 https://github.com/LangilleLab/microbiome_helper/wiki/PICRUSt-tutorial

## Supplementary Material

FIGURE S1 Heatmap of microbial function pathways dynamics in untreated and Lp-treated wheat silages. Colors reflect relative abundance from low (blue) to high (yellow).

FIGURE S2 PICRUSt prediction of functional profiling of the microbial communities based on the 16S rRNA gene sequences. Extended error bar plot indicating differences (0.001%) in the abundance of functional profiles at day 90, in untreated and *L. plantarum* treated silages. The colored circles denote the difference in mean proportion of a particular pathway that is higher in Lp-treated silage (orange), or in untreated silage (blue).

FIGURE S3 PICRUSt prediction of functional profiling of the microbial communities based on the 16S rRNA gene sequences. Extended error bar plot indicating differences in functional profiles of the day 90, untreated and *L. buchneri-*treated silage. The difference higher than 0.001% are presented; the red dots on difference in mean denotes that particular pathway abundance is higher in Lb-treated silage while blue dots denotes it is higher in untreated silage.

TABLE S1 Weighted UniFrac analysis of bacterial community composition natural, *L. plantarum* (Lp)- and *L. buchneri* (Lb)-supplemented (treated) silages during ensiling.

TABLE S2 Relative abundance (%) of bacterial genera found in terminal wheat silages.

TABLE S3 Relative abundance (%) of bacterial genera found after 5 days of aerobic exposure of mature wheat silages.

TABLE S4 Pathways predictions during ensiling of wheat silage with and without *L. plantarum* supplement.
