# Supplementary figures and images for: Bacterial Dynamics of Wheat Silage
Source: Front Microbiol. 2019 Jul 9;10:1532. doi: 10.3389/fmicb.2019.01532 (PMC6632545; doi:10.3389/fmicb.2019.01532)

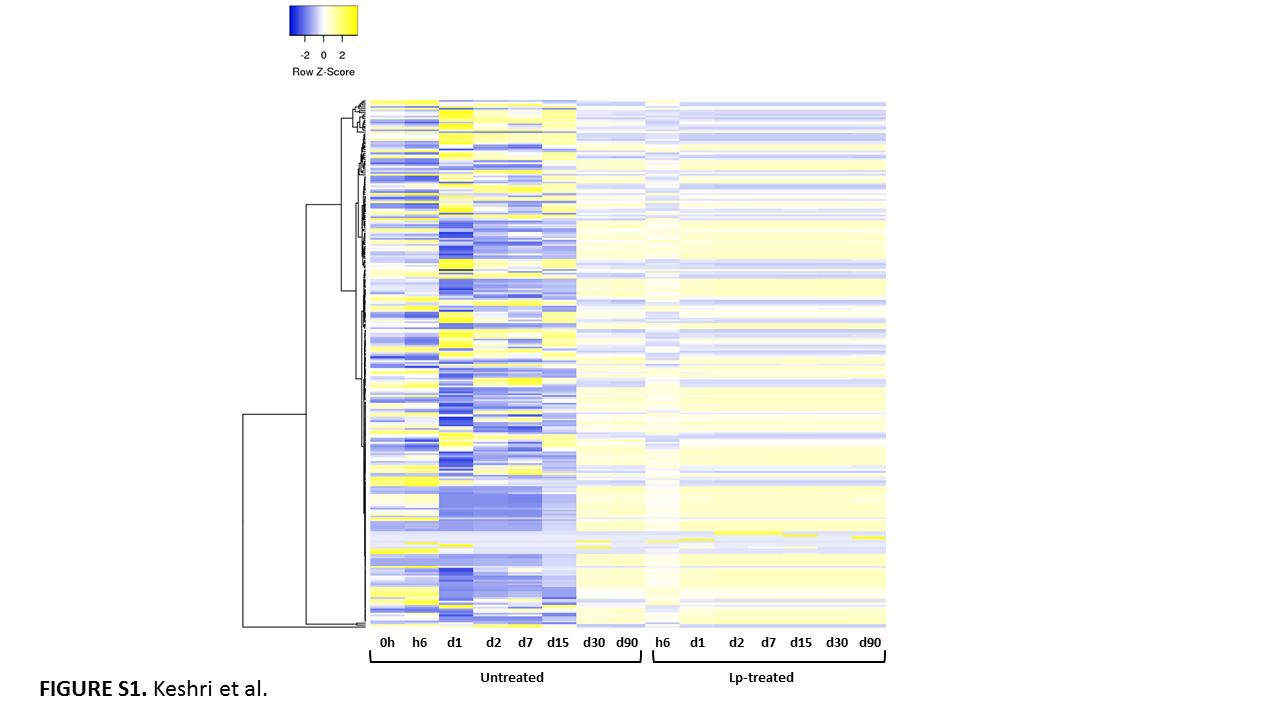

Supplement: FIGURE S1 — Heatmap of microbial function pathways dynamics in untreated and Lp-treated wheat silages. Colors reflect relative abundance from low (blue) to high (yellow). [file Image_1.TIF]

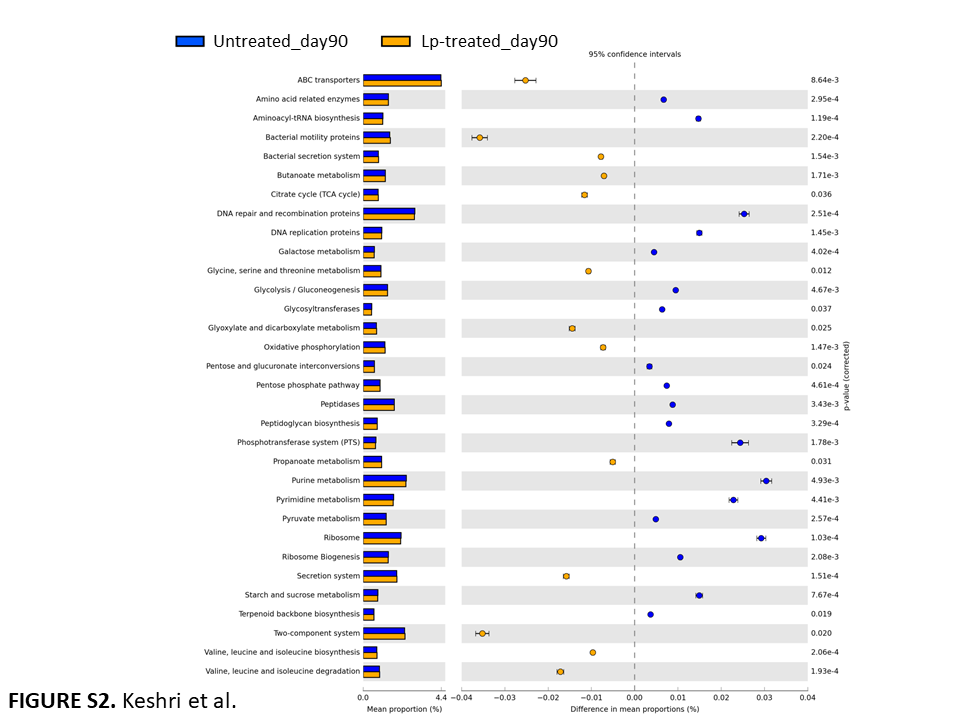

Supplement: FIGURE S2 — PICRUSt prediction of functional profiling of the microbial communities based on the 16S rRNA gene sequences. Extended error bar plot indicating differences (0.001%) in the abundance of functional profiles at day 90, in untreated and L. plantarum treated silages. The colored circles denote the difference in mean proportion of a particular pathway that is higher in Lp-treated silage (orange), or in untreated silage (blue). [file Image_2.TIF]

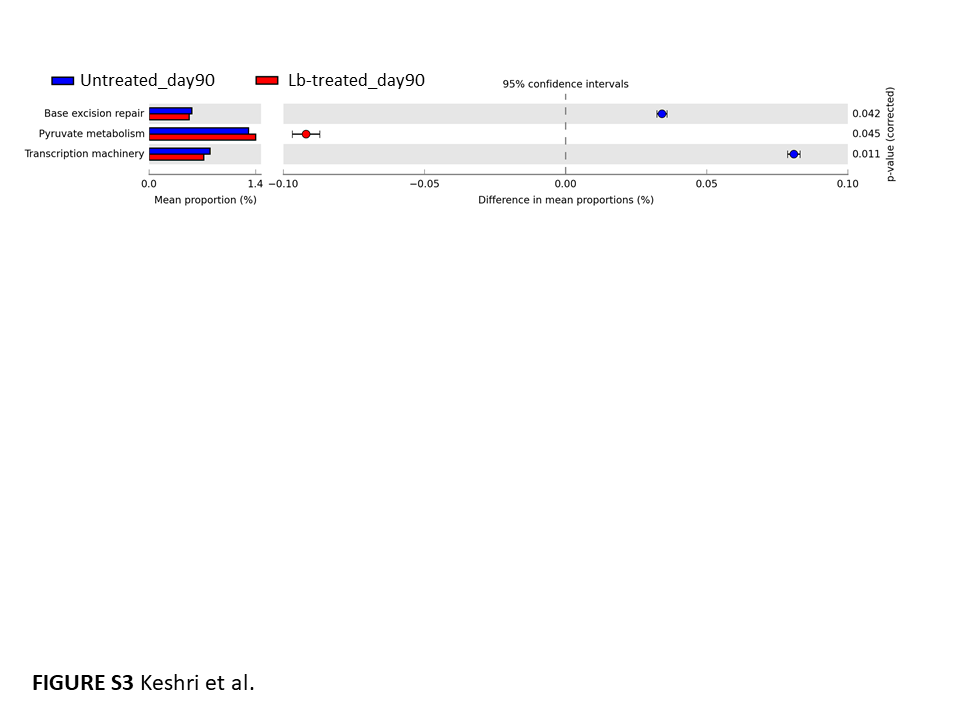

Supplement: FIGURE S3 — PICRUSt prediction of functional profiling of the microbial communities based on the 16S rRNA gene sequences. Extended error bar plot indicating differences in functional profiles of the day 90, untreated and L. buchneri-treated silage. The difference higher than 0.001% are presented; the red dots on difference in mean denotes that particular pathway abundance is higher in Lb-treated silage while blue dots denotes it is higher in untreated silage. [file Image_3.TIF]
