# Supplementary material for: Bacterial Dynamics of Wheat Silage
Source: Front Microbiol. 2019 Jul 9;10:1532. doi: 10.3389/fmicb.2019.01532 (PMC6632545; doi:10.3389/fmicb.2019.01532)
Supplement: TABLE S1 — Weighted UniFrac analysis of bacterial community composition natural, L. plantarum (Lp)- and L. buchneri (Lb)-supplemented (treated) silages during ensiling. [file Table_1.DOCX]

***Supplementary materials***

**Bacterial Dynamics of Wheat Silage**

Jitendra Keshri, Yaira Chen, Riky Pinto, Yulia Kroupitski, Zwi G. Weinberg, Shlomo Sela (Saldinger)*

Department of Food Quality and Safety, Institute for Postharvest and Food Sciences, The Volcani Center, Agriculture Research Organization, Rishon-LeZion, Israel

*Correspondance: **Shlomo Sela (Saldinger)**

[shlomos@volcani.agri.gov.il](mailto:shlomos@volcani.agri.gov.il)

Microbial Food-Safety Research Unit

Department of Food Quality & Safety

Institute for Postharvest and Food Sciences

The Volcani Center, ARO

Derech HaMaccabim Road 68, POB 15159,

Rishon-LeZion 7528809, Israel

**TABLE S1.** Weighted UniFrac analysis of bacterial community composition natural, *L. plantarum* (Lp)- and *L. buchneri* (Lb)- supplemented (treated) silages during ensiling.

| \| **Test of differences between samples** \| **BCC_WSig** \| \| --- \| --- \| \| Untreated_0h versus Untreated_6h \| 0.432 \| \| Untreated_6h versus Untreated_d1 \| 0.069 \| \| Untreated_d1 versus Untreated_d2 \| 0.026 \| \| Untreated_d2 versus Untreated_d7 \| 0.034 \| \| Untreated_d7 versus Untreated_d15 \| 0.01 \| \| Untreated_d15 versus Untreated_d30 \| 0.018 \| \| Untreated_d30 versus Untreated_d90 \| 0.05 \| \| Untreated_d90 versus Untreated_AS \| 0.094 \| \| Untreated_0h versus Untreated_d90 \| <0.001 \| \| Untreated_0h versus Lp-treated_6h \| <0.001 \| \| Lp-treated_6h versus Lp-treated_d1 \| <0.001 \| \| Lp-treated_d1 versus Lp-treated_d2 \| <0.001 \| \| Lp-treated_d2 versus Lp-treated_d7 \| <0.001 \| \| Lp-treated_d7 versus Lp-treated_d15 \| <0.001 \| \| Lp-treated_d15 versus Lp-treated_d30 \| <0.001 \| \| Lp-treated_d30 versus Lp-treated_d90 \| <0.001 \| \| Lp-treated_0h versus Lp-treated_d90 \| 0.004 \| \| Lp-treated_d90 versus Lp-treated_AS \| 0.011 \| \| Untreated_0h versus Lb-treated_d90 \| <0.001 \| \| Lb-treated_d90 versus Lb-treated_AS \| <0.001 \| \| Lp-treated_d90 versus Untreated_d90 \| <0.001 \| \| Lb-treated_d90 versus Untreated_d90 \| <0.001 \| \| Lb-treated_d90 versus Lp-treated_d90 \| <0.001 \| \| Lp-treated_6h versus Untreated_6h \| 0.246 \| \| Lp-treated_d1 versus Untreated_d1 \| <0.001 \| \| Lp-treated_d2 versus Untreated_d2 \| 0.003 \| \| Lp-treated_d7 versus Untreated_d7 \| <0.001 \| \| Lp-treated_d15 versus Untreated_d15 \| 0.007 \| \| Lp-treated_d30 versus Untreated_d30 \| <0.001 \| |  |
| --- | --- | --- | --- | --- | --- | --- | --- | --- | --- | --- | --- | --- | --- | --- | --- | --- | --- | --- | --- | --- | --- | --- | --- | --- | --- | --- | --- | --- | --- | --- | --- | --- | --- | --- | --- | --- | --- | --- | --- | --- | --- | --- | --- | --- | --- | --- | --- | --- | --- | --- | --- | --- | --- | --- | --- | --- | --- | --- | --- | --- | --- |

**TABLE S2.** Relative abundance (%) of bacterial genera found in terminal wheat silages.

| **Genera** | **Untreated_d90** | **Lp-treated_d90** | **Lb-treated_d90** |
| --- | --- | --- | --- |
| *Lactobacillus* | 59.506 | 92.459 | 98.196 |
| *Weissella* | 31.038 | 4.441 | 0.064 |
| *Pediococcus* | 5.879 | 0.216 | 0.017 |
| *Clostridium_sensu_stricto_12* | 1.601 | 0.046 | 0.782 |
| *Enterococcus* | 1.175 | 0.277 | 0.165 |
| *Pantoea* | 0.022 | 0.165 | 0.067 |
| *Raoultella* | 0 | 0.187 | 0 |
| *Massilia* | 0 | 0.049 | 0.124 |
| *Sphingomonas* | 0.004 | 0.107 | 0.037 |
| *Leuconostoc* | 0.099 | 0.008 | 0.024 |
| *Tumebacillus* | 0 | 0.073 | 0 |
| *Janthinobacterium* | 0 | 0.017 | 0.044 |
| *Duganella* | 0 | 0.041 | 0.016 |
| *Rhizobium* | 0.001 | 0.004 | 0.050 |
| *Chryseobacterium* | 0 | 0.046 | 0.001 |
| *Peptoclostridium* | 0 | 0.043 | 0.001 |
| *Clostridium_sensu_stricto_1* | 0.001 | 0.033 | 0.007 |
| *Methylobacterium* | 0.005 | 0.017 | 0.015 |
| *Flavobacterium* | 0 | 0.024 | 0 |
| *Neorhizobium* | 0.005 | 0.014 | 0.005 |
| *Rhodococcus* | 0 | 0.019 | 0.004 |
| *Paenibacillus* | 0 | 0.016 | 0.002 |
| *Buttiauxella* | 0.014 | 0 | 0.002 |
| *Bacillus* | 0 | 0.012 | 0 |
| *Lactococcus* | 0.005 | 0.005 | 0.001 |
| *Epilithonimonas* | 0 | 0.011 | 0 |
| *Sphingobacterium* | 0 | 0.011 | 0 |
| *Psychrobacter* | 0 | 0.008 | 0.002 |
| *Aureimonas* | 0 | 0.008 | 0.001 |
| *Klebsiella* | 0 | 0.008 | 0 |
| *Yersinia* | 0.007 | 0 | 0 |
| *Paracocccus* | 0 | 0 | 0.006 |
| *Xanthomonas* | 0 | 0.004 | 0.001 |
| *Staphylococcus* | 0 | 0.004 | 0 |
| *Citrobacter* | 0.004 | 0 | 0 |
| *Psychrobacillus* | 0.001 | 0 | 0.002 |
| *Jeotgalicoccus* | 0 | 0 | 0.003 |
| *Aerococcus* | 0.003 | 0 | 0 |
| *Devosia* | 0.001 | 0 | 0.001 |
| *Garciella* | 0 | 0 | 0.002 |
| *Lachnoclostridium_5* | 0 | 0 | 0.002 |
| *Turicibacter* | 0 | 0 | 0.002 |
| *Providencia* | 0 | 0 | 0.002 |
| *Hymenobacter* | 0 | 0 | 0.002 |
| *Hafnia* | 0 | 0 | 0.002 |
| *Carnobacterium* | 0.001 | 0 | 0 |
| *Desemzia* | 0.001 | 0 | 0 |
| *Pleurocapsa* | 0 | 0 | 0.001 |
| *Roseomonas* | 0 | 0 | 0.001 |
| *Escherichia-Shigella* | 0 | 0 | 0.001 |
| *Pseudomonas* | 0 | 0 | 0.001 |
| *Unclassified* | 0.626 | 1.630 | 0.349 |

Untreated denotes wheat silage ensiled with natural microbiome, Lp-treated denotes wheat silage ensiled with *L. plantarum* supplement and Lb-treated denotes wheat silage ensiled with *L. buchneri* supplement. d90 denotes 90 days of ensiling in anaerobic conditions.

**Table S3.** Relative abundance (%) of bacterial genera found after 5 days of aerobic exposure of mature wheat silages.

| **Genera** | **AST_Untreated** | **AST_Lp-treated** | **AST_Lb-treated** |
| --- | --- | --- | --- |
| *Lactobacillus* | 85.922 | 49.832 | 98.414 |
| *Clostridium_sensu_stricto_1* | 0 | 45.328 | 0.616 |
| *Weissella* | 12.799 | 3.193 | 0.100 |
| *Enterococcus* | 0.519 | 0.261 | 0.224 |
| *Clostridium_sensu_stricto_12* | 0.354 | 0.118 | 0.013 |
| *Bacillus* | 0 | 0.245 | 0.003 |
| *Pantoea* | 0.022 | 0.026 | 0.061 |
| *Buttiauxella* | 0.007 | 0.038 | 0.055 |
| *Leuconostoc* | 0.046 | 0.012 | 0.031 |
| *Sphingomonas* | 0.003 | 0.036 | 0.009 |
| *Pediococcus* | 0.020 | 0.012 | 0.009 |
| *Paenibacillus* | 0 | 0.032 | 0 |
| *Romboutsia* | 0 | 0.028 | 0 |
| *Massilia* | 0 | 0 | 0.026 |
| *Lactococcus* | 0.004 | 0.014 | 0 |
| *Serratia* | 0.001 | 0.014 | 0 |
| *Stenotrophomonas* | 0.015 | 0 | 0 |
| *Intestinibacter* | 0 | 0.014 | 0 |
| *Jeotgalicoccus* | 0 | 0 | 0.011 |
| *Lachnoclostridium_5* | 0 | 0.009 | 0 |
| *Neorhizobium* | 0.004 | 0 | 0.004 |
| *Methylobacterium* | 0 | 0 | 0.008 |
| *Sphingobacterium* | 0.004 | 0 | 0.003 |
| *Duganella* | 0 | 0 | 0.007 |
| *Oceanobacillus* | 0 | 0 | 0.003 |
| *Psychrobacter* | 0 | 0 | 0.003 |
| *Pseudomonas* | 0 | 0 | 0.003 |
| *Aerococcus* | 0.003 | 0 | 0 |
| *Enterobacter* | 0.003 | 0 | 0 |
| *Flavobacterium* | 0 | 0 | 0.002 |
| *Turicibacter* | 0 | 0 | 0.002 |
| *Rathayibacter* | 0 | 0 | 0.002 |
| *Peptoclostridium* | 0 | 0 | 0.001 |
| *Roseomonas* | 0 | 0 | 0.001 |
| *Rheinheimera* | 0 | 0 | 0.001 |
| *Rhodococcus* | 0 | 0 | 0.001 |
| *Exiguobacterium* | 0 | 0 | 0.001 |
| *Devosia* | 0 | 0 | 0.001 |
| *Paracocccus* | 0 | 0 | 0.001 |
| *Unclassified* | 0.273 | 0.786 | 0.381 |

Untreated denotes wheat silage ensiled with natural microbiome, Treated_LP denotes wheat silage ensiled with *L. plantarum* supplement and Treated_LB denotes wheat silage ensiled with *L. buchneri* supplement. AST denotes Aerobic stability test i.e., aerobic exposure of mature silages for 5 days.
